# Medically induced trauma and compassion: Reflections from the sharp end of care

**DOI:** 10.4103/0019-5049.68368

**Published:** 2010

**Authors:** Frederick van Pelt

**Affiliations:** Director, Global Programs, Partners Harvard Medical International, Boston, USA. E-mail: fvanpelt@phmi.partners.org

## INTRODUCTION

Since the Institute of Medicine’s landmark report ‘To Err is Human’ in 1999, wherein it has been estimated that up to 100,000 patient lives are lost each year in the United States due to medical error, healthcare in the United States as well as globally has invested great effort and resources in the improvement of quality and patient safety. Although research and reporting is typically focused on process improvement methodologies and the changes being implemented in healthcare delivery, very little attention has been focused on the management of adverse medical events, specifically the impact that these events have on patients, families and care providers. It would be a rare care provider who has not been directly involved in an adverse medical event or who has not witnessed a colleague having been involved in an adverse event. Most adverse medical events are shrouded in secrecy and gaining the opportunity to share the experience in an open and meaningful way is extremely difficult.

I would like to share with you a personal account of an adverse medical event that I experienced approximately ten-and-a-half years ago, as an anaesthesiologist. It is my hope that sharing this story will bring to the surface those buried adverse events that the readers have experienced and that an opportunity might be created to enable a careful revisiting of the impact that these events may have on all of our professional and personal lives. By the end of this editorial I hope that you will come away with four takeaways:

1. In spite of our best efforts to improve quality and safety, adverse medical events will always occur
2. Adverse events have a significant impact on patients, families and, importantly, care providers
3. There are many potential causes for us to consider why we manage adverse events the way we do
4. Our management of adverse events is but the visible tip of the way we manage our everyday practice and personal lives

## THE EVENT

On November 18, 1999, I was providing anaesthetic care for a 37-year-old woman undergoing a total ankle replacement. Without deviating from the standard of care, I placed a popliteal fossa nerve block pre-operatively with bupivicaine. As we were preparing to go to the operating room, the patient experienced a grand mal seizure, which rapidly progressed to cardiac arrest. After approximately 10 minutes of intense resuscitation the patient remained unresponsive and the situation was dire. A fully prepped cardiac operating room was fortuitously available and the patient was rushed into the room, where she underwent a midline sternotomy for emergent cardiopulmonary bypass. The patient’s cardiac rhythm was restored and after being weaned off the bypass machine she was taken, intubated, to the cardiac intensive care unit.

The event itself was dramatic and it was only teamwork, professionalism and the synchronous availability of resources that allowed us to perform this miraculous save. It was not until the resuscitation had successfully ended that I started to appreciate a very odd response to this event by all of the team members: the emotional detachment was persisting.

Although I was strongly advised not to venture, I went to speak to the patient’s husband about the event and it was only then that the full impact of what had just occurred hit me. The full force of the emotional and physical energy that was directed towards me by the husband shattered any sense of professional distance that I had been able to maintain (and had been taught to maintain), and all of a sudden I was faced with the real impact that adverse events have on families as well as, uncomfortably, myself. The operating room had been cleaned, there were cases to be done and everyone was going about their business. I was sent home for the day and returned to work the next day with a regular caseload — the adverse event had seemingly vanished for everyone but me.

The patient remained hospitalized for approximately ten days and fortunately progressed to full recovery. My sense of responsibility and my exposure to the emotional impact compelled me to seek a visit with the patient while she was in hospital. However, this was not to be as I was actively and repeatedly prevented from having any contact with the patient. The patient had only been told that she had experienced an allergic reaction to the anaesthetic without further discussion, and was discharged home with post-sternotomy wound care instructions and with a follow-up appointment for a visiting nurse.

## THE AFTERMATH

I had been completely isolated from having any meaningful outlet about what had happened and faced a situation where it appeared that I would be unable to ever establish any direct contact with the patient. However, I could not compromise my personal integrity; I had to do the right thing and reach out to the patient. Without informing the hospital, I wrote a letter to the patient, in which I acknowledged the emotional impact that this event had had on her family as well as on myself, I apologized for my responsibility for having initiated this sequence of events, and I invited the patients to open communication if and when she was willing and interested.

The patient, Linda Kenney, contacted me by telephone approximately six months after the event occurred. After sharing with her all the details of the event, the conversation finished with the patient offering me forgiveness. It was one of the most powerful and uplifting experiences in my life and served to set my life in an entirely new direction. In an instant, the burden that I had been carrying on my chest had lifted and I was free.

Our joint realization of the profound need for awareness and support around adverse medical events resulted in the creation of a not-for-profit foundation, Medically Induced Trauma Support Services, to provide neutral access to support for patients, families and care providers impacted by medical events (see www.MITSS.org). Furthermore, it catalysed the creation of a hospital-based peer support network for care providers at my hospital.[1] To our great surprise, these two initiatives that had blossomed out of a tragedy transformed by transparency and apology were all of a sudden propelled to national and international attention. We had awakened a sleeping giant in healthcare quality and safety that could no longer be ignored.

## REFLECTIONS

There were a number of system failures for the clinician. These included: the absence of any adverse event debriefing; the absence of guidance or advocacy during the quality assurance, peer review or root cause analysis process; a complete absence of emotional support; and no system to advocate or support any form of communication with the patient. The system’s failures for the patient and family included: minimal disclosure about what happened in the hospital; limited family support in the hospital; no support after discharge; and no follow-up communication.

With a broken process of adverse event management such as this, it is very clear that the impact is not understood and the learning from these experiences is not captured. The result is angry patients and families, isolated clinicians where morale and job performance are impacted and where a baseline of relatively poor communication among colleagues becomes the norm. Furthermore, the absence of support and the silence around adverse medical events creates great difficulty in assessing what actually precipitates these events, which results in sub-optimal system improvements. This is a vicious cycle that continually repeats and builds on itself.

Therefore the question arises: if we are in a profession where our charge is to provide compassionate care to patients, why is it that when adverse events occur this guiding principle suddenly evaporates? In healthcare globally, the clinician is viewed as ‘superhuman.’ Although care providers are exposed to adverse medical events on a daily basis, taking responsibility for these events is unacceptable within the healthcare profession. Indeed, this perception is reinforced in the Hippocratic Oath, ‘first do no harm.’ This often leads to a sense of isolation, shame and humiliation as patient, families and colleagues berate and distance themselves from the involved care provider.

The second element to consider is the process of ‘numbing down.’ During our training care providers are taught to distance themselves emotionally from our patients through empathy. That is to say, understand and acknowledge how our patients feel without becoming personally involved. With time, many empathetic care providers become experts at suppressing their ability to feel, and the compassion that initially attracted care providers to healthcare is supplanted with detached professional competence. This process of numbing down blunts the ability of the caregiver to understand and acknowledge the emotional impact that adverse events have on patients, families and themselves. In addition, if disclosure and apology are offered to the patient and / or family members, the detached caregiver may lack the authenticity that is critical during this kind of conversation and quickly erode the crucial bond of trust between patient, family and care provider.

A third element to consider is the absence of system-based thinking. It is well known in principle and in practice that greater than 90% of the results experienced in healthcare are functions of the systems in which care providers work, rather than their individual efforts. The systems that compromise the quality of care are for the most part invisible to the care providers. Thus, the root cause of the process breakdown resulting in adverse events is never addressed. Rather, the care providers are blamed for ‘mistakes’ that are out of their control rather than looking first at the less than optimal systems as the source of the poor outcomes and inefficiency.

One of the most striking revelations for me, in this experience, is how modern healthcare has separated the science of medicine from the art of healing. It is well known today as well as historically that medicine is a combination of both. The scientific approach has provided us with a great understanding of the disease with new treatment modalities; however, many disease processes and illnesses remain unresponsive to our latest interventions. By contrast, we have also experienced patients with seemingly incurable conditions miraculously turn the corner, defying our scientific knowledge and prognoses. The modern medical scientist will attribute this to luck or good fortune. However, it seems to me that there is a deeper message that we need to revisit and to reacquaint ourselves with.

It would appear that as we embrace the ever advancing medical technology, a clinical approach is emerging that is mechanical and promotes lesser personal contact. As a result, we are increasingly treating our patients as diseases and procedures and our colleagues as functionaries rather than as human beings. The great physicians and healers of the past and present are able to incorporate the best scientific knowledge with the intangible healing touch for their patients. Whether conscious or unconscious, it is the ability of the great physician or healer to blend the two seamlessly and to recognise that patients are human beings first and that illness and suffering are best treated using both modalities. In some cases it is the scientific side that appears to provide the greatest benefit; other times it appears that it is the compassionate touch, which restores health.

How we relate to our patients and their families is also directly linked to how we treat each other as colleagues. We might ask ourselves whether we are treating each other as human beings, recognising that both the factual and the intangible must be present if we are to truly respect and relate to each other. It is when we can see each other as human beings and break down the barriers of individualism, competition and fear that compassion emerges. Compassion begins with self and then extends to colleagues and patients. It should be the foundation of all of our relationships, not just when catastrophe strikes. Perhaps it is the absence of compassion in our daily lives that explains why we have such great difficulty in managing adverse events.

The absence of compassion in our daily routines, and particularly when things go wrong, precludes us from doing the right thing. Doing the right thing is not formulaic and does not stem from knowledge or intellect. Rather it is the expression of intelligence and wisdom. The emergence of intelligence and compassion are the result of awareness, integrity, commitment and perhaps most importantly authenticity. It is through allowing ourselves to become vulnerable, to become human and to listen that great possibility and transformation results. This applies to us not only professionally, but also in our personal lives.

Patients and families expect four things following adverse events:

1. Transparent communication about the event as information becomes available
2. An apology or an acknowledgment of the event’s impact
3. Reassurance that an organisational response to prevent recurrence is underway
4. Individualised support

I would submit that the impacted care providers expect these as well. As Gandhi’s timeless quote, ‘be the change you want to see in the world’ suggests, it is the ‘human being’ rather than the ‘human doing,’ which will ultimately create sustained transformation in healthcare as well as in our personal lives.
